# Can a tailored exercise and home hazard reduction program reduce the rate of falls in community dwelling older people with cognitive impairment: protocol paper for the i-FOCIS randomised controlled trial

**DOI:** 10.1186/1471-2318-14-89

**Published:** 2014-08-15

**Authors:** Jacqueline CT Close, Jacqueline Wesson, Catherine Sherrington, Keith D Hill, Sue Kurrle, Stephen R Lord, Henry Brodaty, Kirsten Howard, Laura N Gitlin, Sandra D O’Rourke, Lindy Clemson

**Affiliations:** 1 Neuroscience Research Australia, University of New South Wales, PO Box 1165, Randwick, NSW 2031, Australia; 2 Prince of Wales Clinical School, Sydney, UNSW, Australia; 3 Ageing Work and Health Research Unit, Faculty of Health Sciences, The University of Sydney, PO Box 170, Lidcombe, NSW 2141, Australia; 4 The George Institute for Global Health, The University of Sydney, PO Box M201, Missenden Rd, Sydney, NSW 2050, Australia; 5 School of Physiotherapy and Exercise Science, Curtin University, GPO Box U1987, Perth, WA 6845, Australia; 6 Department of Rehabilitation and Aged Care, Hornsby Hospital, Hornsby, NSW 2077, Australia; 7 Centre for Healthy Brain Ageing, Sydney, UNSW, Australia; 8 Sydney School of Public Health, The University of Sydney, A27 Edward Ford Bld, Sydney, NSW 2006, Australia; 9 Schools of Nursing and Medicine, Center for Innovative Care in Aging, Johns Hopkins University, Baltimore, MD, USA

**Keywords:** Cognitive impairment, Dementia, Accidental falls, Prevention, Intervention

## Abstract

**Background:**

The rate of falls in community dwelling older people with cognitive impairment (CI) is twice that of a cognitively intact population, with almost two thirds of people with CI falling annually. Studies indicate that exercise involving balance and/or a home hazard reduction program are effective in preventing falls in cognitively intact older people. However the potential benefit of these interventions in reducing falls in people with CI has not been established.

This randomised controlled trial will determine whether a tailored exercise and home hazard reduction program can reduce the rate of falls in community dwelling older people with CI. We will determine whether the intervention has beneficial effects on a range of physical and psychological outcome measures as well as quality of life of participants and their carers. A health economic analysis examining the cost and potential benefits of the program will also be undertaken.

**Methods and design:**

Three hundred and sixty people aged 65 years or older living in the community with CI will be recruited to participate in the trial. Each will have an identifiable carer with a minimum of 3.5 hours of face to face contact each week.

Participants will undergo an assessment at baseline with retests at 6 and 12 months. Participants allocated to the intervention group will participate in an exercise and home hazard reduction program tailored to their cognitive and physical abilities.

The primary outcome measure will be the rate of falls which will be measured using monthly falls calendars. Secondary outcome measures will include the risk of falling, quality of life, measures of physical and cognitive function, fear of falling and planned and unplanned use of health services. Carers will be followed up to determine carer burden, coping strategies and quality of life.

**Discussion:**

The study will determine the impact of this tailored intervention in reducing the rate of falls in community dwelling older people with CI as well as the cost-effectiveness and adherence to the program. The results will have direct implications for the design and implementation of interventions for this high-risk group of older people.

**Trial registration:**

The protocol for this study is registered with the Australian New Zealand Clinical Trials Registry - ACTRN12614000603617

## Background

Cognitive impairment (CI), including dementia, has and will continue to have an enormous impact on society. In 2010, dementia was the third leading cause of death in Australia, the second leading cause of burden of disease and the leading cause of disability. Similarly, falls and fall related injury in older people continue to challenge health and social care systems on a worldwide basis. The rate of falls in community dwelling older people with dementia is twice that of a cognitively intact population with almost two thirds of people with dementia falling annually [1,2]. Older people with dementia have a four-fold increased risk of hip fracture and a three-fold increased risk of 6-month mortality following a fracture when compared to older people without dementia. They are also more likely to enter residential aged care as a result of a fall related injury [3-5].

Exercise and home hazard reduction programs are two of the most effective interventions for preventing falls in cognitively intact older people [6-8]. A systematic review has reported a 17% reduction in falls across 44 studies of exercise as a single intervention (pooled rate ratio 0.83, 95% CI 0.75 - 0.91) with the greatest effect (pooled rate ratio 0.58, 95% CI 0.48 - 0.69) from programs that provided a high level balance challenge and of sufficiently high prescribed exercise dose [7]. A meta-analysis examining the efficacy of home safety interventions indicated the strongest fall prevention effects in those identified at increased risk of falls (pooled rate ratio 0.61, 95% CI 0.47 - 0.79) [8,9].

The trials included in the above systematic reviews have largely excluded people with CI and only one large, adequately powered randomized controlled trial has been conducted that has specifically included people with CI [10]. This study tested interventions previously trialed in cognitively intact older people without adapting them to the cognitive abilities of an impaired population. No reduction in falls was evident in the intervention group compared to the control group (relative risk 0.92, 95% CI 0.81-105) suggesting the simple application of an intervention shown to work in cognitively intact populations does not prevent falls in cognitively impaired older adults.

There is now strong evidence that fall risk is increased in older people with CI as a result of both a) exacerbation of risk factors found in cognitively intact older people and b) fall risk factors that are specific to CI. A prospective cohort study of risk factors for falls in 177 older community dwelling people with CI found a high rate of falls in this group with 62% of participants falling in the follow-up year [11]. Measures which were significantly associated with falls in univariate analyses included poor visuospatial skills, reduced executive function, presence of depression and anxiety, poor balance, slow reaction time, reduced functional mobility and use of psychotropic medications. The final multivariate model showed tests of standing (IRR 2.28, 95% CI 1.54 – 3.36) and leaning balance (IRR 1.78, 95% CI 1.20 – 2.62) and a measure of depressive symptomatology (IRR 2.31, 95% CI 1.59 – 3.36) to be significant and independent predictors of falling. Conclusions drawn from this and other work [12,13] suggest that a) an exercise intervention aimed at improving balance and reducing depressive symptoms has the potential to reduce falls in older people with CI, b) an occupational therapy intervention comprising environmental hazard reduction and instructions regarding safe transfers and mobility in the home context is highly relevant due to the high proportion of home falls suffered by this group and c) the presence of the CI is likely to influence the safe and effective delivery of interventions.

There is now good evidence that a CI specific approach to care can be effective in improving daily function for the participant and sense of competence for the carer [14,15]. Using this approach, interventions are based on the individuals’ preserved cognitive abilities and the caregiver is provided with the skills to work effectively with the person with CI.

The current randomised controlled trial will examine whether such an individually tailored, CI specific approach to the delivery of an exercise and home hazard reduction program can reduce the rate of falls in cognitively impaired older people.

## Methods

### Design

**Figure 1:**
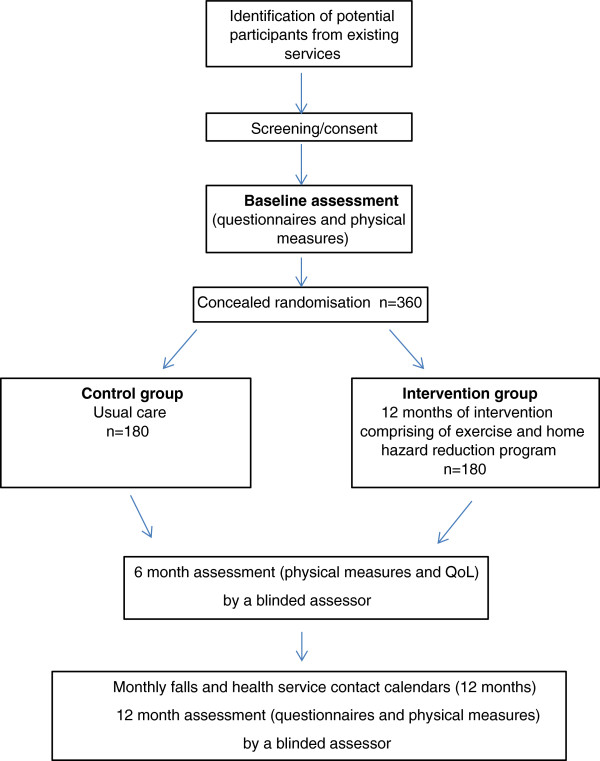
Flow of participants through the study.

A randomised controlled trial will be conducted with 360 older people with cognitive impairment. Figure 1 gives an overview of the study design.

Trial reporting will be guided by the CONSORT Statement extensions for trials of non-pharmacological interventions and pragmatic intervention trials.

### Participants

Participants will be recruited via two centres in Sydney, Australia. To be eligible for the study, participants must be a) community dwelling, b) aged 65 years or older, c) have CI as defined by having a Mini-Mental State Examination (MMSE) score or Mini-Addenbrookes Cognitive Examination Australian Version (Mini-ACE) <24, an Addenbrookes Cognitive Examination – III, Australian Version (ACE-III) <83 or a specialist clinical diagnosis of cognitive impairment, d) have an identifiable and consenting “person responsible” and e) have a carer who has a minimum of 3.5 hours of face to face contact with the participant each week and is willing to assist with reporting falls and supervising the exercise intervention (3 or more times per week).

People living in Residential Aged Care Facilities will be excluded as will those with an MMSE or Mini-ACE < 12/30 as it is considered unlikely such people will be able to engage with the intervention. Participants need to have sufficient English language skills to understand the assessment and intervention procedures. Other exclusion criteria include inability to walk more than one metre despite assistance with a walking aid and/or another person, blindness, severe psychiatric disease, a progressive neurological disease other than dementia or any medical condition precluding exercise (e.g. unstable cardiac disease).

Ethics approval has been obtained from the relevant human research ethics committees (South Eastern Sydney Local Health District Ethics committee (HREC 14/046), Northern Sydney Area Health Service and Neuroscience Research Australia).

### Measurement and procedures

All eligible participants will be visited at home by a research physiotherapist. The study will be explained in detail to the participant and their carer and written consent will be obtained prior to the baseline assessment. Capacity to consent will be determined by whether the potential participant demonstrates an ability to comprehend, retain and recall information about the study and any risks involved. Where there is doubt about a potential participant’s ability to process, retain and recall information about the study, assent to participate will be obtained from the identified “person responsible”.

All participants will be assessed in their home at three time points: on entry to the study (baseline assessment, prior to randomisation); at 6 months post randomisation and at 12 months post randomisation. Table 1 highlights the measures to be undertaken at each time point. Post randomisation assessments will be undertaken by assessors blinded to group allocation.

**Table 1:** List of measures collected at baseline (BA), 6 month (6A), and 12 month assessments (12A) for all study participants

**Information collected for all participants**	**BA**	**6A**	**12A**	**O**
**Socio-demographics**				
**Age, gender, marital status, education, occupation, place and type of residence and number of co-habitants**	Y	N	N	N
**General health and function**				
Disease history, medication use, assistive walking device and detailed information on previous falls and fractures	Y	N	N	N
The Incidental and Planned Exercise Questionnaire (IPEQ) will provide estimates of the frequency and duration of planned and casual day-to-day activities [16]	Y	N	Y	S
Disability Assessment for Dementia to assess everyday functioning [17]	Y	N	Y	S
**Quality of life**				
The EQ5D-5 L is a widely used utility-based quality of life instrument for estimating QALYs for economic evaluations [18]	Y	Y	Y	S
**Neuropsychological**				
Fear of falling will be assessed using Icon-FES. The scale has excellent reliability, validity for people with CI, and responsiveness-to-change [19]	Y	N	Y	S
The 15-item Geriatric Depression Scale will assess symptoms of depression [20]	Y	N	Y	S
The 9-item Goldberg Anxiety Scale will assess symptoms of generalised anxiety [21]	Y	N	Y	S
Frontal Assessment Battery [22]	Y	N	Y	S
Addenbrooke’s Cognitive Examination (ACE-III) [23]	Y	N	Y	S
**Physical measures**				
The Physiological Profile Assessment (measures visual contrast sensitivity, proprioception, quadriceps strength, simple reaction time, and postural sway while standing on a foam rubber mat with eyes open) [24].	Y	Y	Y	S
Short Physical Performance Battery [25]	Y	Y	Y	S
The Maximal Balance Range test (assesses the ability to lean as far forward and backwards as possible)	Y	Y	Y	S
The Coordinated Stability test (assesses the body position in a steady and coordinated manner near the limits of their base of support).	Y	Y	Y	S
**Carer interview and questionnaires**				
Carer burden will be assessed with the Zarit Burden of Care Index [26]	Y	N	Y	S
The EQ5D-5 L for estimating QALYs for economic evaluations [18]	Y	Y	Y	S
The Montreal Cognitive Assessment (MoCA) [27] will be administered to consenting carers over 65 years	Y	N	N	N
Caregiver skill enhancement will be measured using the Task Management Strategy Index [12]	Y	N	Y	S
**Falls and health service use**				
Falls and fall related injuries (monthly diaries) [28]				P
Planned and unplanned use of health services (monthly diaries)				S

Note: Y = YES, N = NO, BA = Baseline assessment, 6A = 6 month assessment, 12RA = 12 month reassessment.

O = Outcome measure, S = Secondary, P = Primary.

### Randomisation

Participants will be randomised after completion of the baseline assessment. Randomisation will be stratified by hospital recruitment site using computer generated random numbers with variable block sizes of 6–8. The randomisation will be performed centrally using an independent web-based program by an investigator not involved in assessment or intervention.

### Control group

Following baseline assessment, the control group will receive usual care from their medical practitioner and community services. The control group will not receive any additional intervention as part of the study.

### Intervention group

Prior to commencing the intervention, each participant’s medical practitioner will be contacted to ensure support for the participant’s involvement in the study. All participants in the intervention group will undergo an assessment of functional cognition and a home safety assessment to inform the approach to the exercise and home safety program. The intervention group will receive a combined total of 11 visits over a 12 month period from the occupational therapist and physiotherapist, with the ratio of visits determined by the identified needs of the person at the time of assessment and their home environment. Factors influencing the ratio of visits will include physical performance, functional cognition, participant and carer willingness to engage, identified intervention priorities and issues of safety. Additional phone calls in between visits will be used to encourage continued engagement in the intervention and identify any problems with either the exercise or environmental recommendations.

#### ***Functional cognition***

Allen’s Model of Cognitive Disabilities [29] will be used to tailor the delivery of the exercises and home safety program to participants’ cognitive abilities. Participants will be assessed using the standardised performance based Large Allen’s Cognitive Level Screen – 5 (LACLS-5) [30] and the Allen’s Diagnostic Module [31] to provide an estimate of global information processing abilities. Six cognitive levels are hierarchically defined with ordinal modes of performance within each level (level 1 = severe impairment; level 6 = normal functioning). Participants are assigned a level/mode based on motor actions, problem solving skills and cognitive assistance required during assessment tasks. This information will be used to guide the delivery of the intervention so it matches the preserved abilities of each participant.

#### ***Home hazards safety program***

The home environment and the proficiency of participants to function within their home environment will be assessed before commencing the exercise intervention. Home hazards will be identified using the Westmead Home Safety Assessment tool [9] and recommendations will be prioritised according to risk and undertaken in negotiation with the participant and carer. Participants will be provided with a home safety booklet, adapted to their cognitive abilities, outlining recommendations. For example, explicit explanation (with pictures) of why situations may be hazardous will be provided to participants who may not be able to comprehend abstract concepts such as safety. Typical recommendations will include removing/ securing loose mats, highlighting step edges with fluorescent tape and installing sensor lighting to illuminate walkways and bathrooms at night. Grab rails and commonly recommended equipment will be accessed through existing services in the community with participants funding these interventions. Minor home safety measures will be installed free of charge, i.e. tape for securing mats or highlighting step edges.

### Exercise program

The exercise intervention will consist of exercises commonly used in fall prevention research and clinical practice. The program will be delivered by experienced physiotherapists to minimise the risk of adverse events and taught to the carer who will supervise practice sessions as necessary. The visits will be more frequent at the beginning of the program to ensure safety and enable tailoring of the program to the cognitive and physical abilities of each participant. Each scheduled visit will last 40–60 minutes and participants will then be requested to undertake a 30 minute exercise program up to 6 times per week at home for 12 months. The exercises will be primarily conducted in the standing position and emphasise balance training and muscle strengthening.

The optimal intensity and type of exercises for each participant will be assessed and adjusted by the study physiotherapists to ensure that the intervention remains safe but challenging.

Uptake and adherence to the home safety and exercise recommendations will be recorded.

### Safety

As this study involves the prescription of home-based exercise to a high risk population, safety while exercising will be a prime consideration. Participants will be shown how to perform exercises with stable supports if necessary and will be provided with a tailored exercise booklet. This material will be adapted to suit the participants’ cognitive abilities including large photographs of exercises, simple instructions and safety precautions. Carers will be considered intervention partners and will be taught how best to supervise exercise sessions including the type of cueing required.

A logbook for recording exercises completed and effects of exercise (e.g. muscle soreness) will be provided. Participants (or their carers) will be advised to telephone study staff if they experience any major adverse effects, e.g. muscle soreness lasting for more than 48 hours that interferes with daily activities or requires medical attention. If a participant becomes unwell or has an admission to hospital, the program will be resumed when the participant and the relevant professionals deem him/her well enough to participate again, and the exercise program may be modified by the therapist if the participant’s status has changed from pre-hospital.

### Outcome measures

The primary and secondary outcome measures are outlined in Table 1. The primary outcome measure will be the rate of falls over the 12 month follow up period. A fall will be defined using the internationally derived consensus definition of “an unexpected event in which the participant comes to rest on the ground, floor, or lower level” as a result of a loss of balance [28]. *Fall rate* will be calculated from monthly calendars. All participants will receive 12 calendars at the time of the baseline assessment. Participants/carers will be asked to record falls and use of health and community services on the calendars and return the information to the co-ordinating centre each month using prepaid addressed envelopes. Participants/carers who do not return calendars will be telephoned to ask for the information. Participants who report having fallen will be telephoned to seek additional information about the circumstances and consequences of the fall. Staff who receive calendars, make follow up phone calls and enter data will be unaware of participants’ group allocation.

The secondary outcome measures listed in Table 1 will be collected at the identified time points by an assessor unaware of group allocation. The order of measurements will be standardised. Participants/carers will be instructed not to inform the assessor of their intervention status and all home exercise equipment will be removed or concealed prior to the assessment.

The cost of implementing the exercise and home hazard program will be obtained from trial records and NeuRA financial records. The use of public and private healthcare resources will be recorded as part of the monthly calendars over the 12-month study period. Resources will be costed using published government sources where available (Pharmaceutical Benefits Scheme unit costs; Medicare Benefits Schedule, and the National Hospital Costs Data collection). We will also utilise our own previously collected data [32] to provide estimates of out of pocket costs to patient and family.

### Sample size

Power analysis (with 5% significance level, 80% power) has been undertaken using data from our previous work and similar studies. A total of 360 participants (180 per group) will provide 80% power to detect a significant 30% lower rate of falls for intervention participants than control participants (i.e. IRR 0.70). We assumed alpha (measure of over-dispersion in negative binomial regression model) to be 0.8 and the control group rate of falls would be 1.8 falls/person year over the 12-month follow-up period - based on our cohort study [1]. An average follow-up period of 11 months (rather than the planned 12 months) was used in the sample size calculation to account for loss to follow-up.

### Statistical methods

An intention-to-treat approach will be used for all analyses. The number of falls per person-year in the intervention and control groups will be compared with incidence rate ratios using negative binomial regression. This provides a more powerful analysis than a simple comparison of the proportion of fallers in the follow-up period, as it takes into account all falls during the trial, and also the distribution of falls, which is Poisson-like but has a longer tail. General linear models will be used to assess the effect of group allocation on the continuously scored secondary outcome measures. Modified Poisson regression models will be used to compare groups on dichotomous outcome measures. Predictors of adoption and adherence will be analysed using multivariate modelling techniques such as multiple linear and logistic regression.

### Economic analysis

The economic evaluation will be conducted from the perspective of the health and community service provider. Data will be collected regarding costs of the exercise and home hazard reduction program delivery (including staff costs, training, capital costs and consumables). Inpatient hospital admissions and duration, emergency department presentations and other health and community service contact (including GP and specialist visits) will be recorded via the monthly calendars. Pharmaceutical use will also be collected. Incremental cost-effectiveness ratios will be calculated using multiple health outcomes, i.e. the incremental cost per a) fall prevented, b) per fall requiring medical attention avoided, c) ED presentation avoided, d) hospital admission avoided, and e) QALY gained (based on the EQ5D-5 L). Using the mean costs in each trial arm, and the mean health outcomes in each arm, the incremental cost per health outcome (a-e, above) of the intervention group compared to the control group will be calculated and results will be plotted on a cost-effectiveness plane. Bootstrapping will be used to estimate a distribution around costs and health outcomes, and to calculate the confidence intervals around the incremental cost-effectiveness ratios taking account of joint uncertainty in costs and benefits. One way sensitivity analysis will be conducted around key variables; a cost-effectiveness acceptability curve will be plotted. A cost-effectiveness acceptability curve provides information about the probability that an intervention is cost-effective, given a decision maker’s willingness to pay for each additional health outcome gained.

## Discussion

Cognitive impairment (including dementia) is a national and international health priority and the challenges faced with managing the increasing incidence and prevalence of this syndrome are substantial for individuals, health care systems and society more generally. Failing to address the issue of falls and fall related injury in this group has enormous consequences with huge cost implications to both the health and aged care sectors as well as to families and the informal care network more broadly.

Most older people, including those with CI, wish to remain in their own home for as long as possible. By reducing the rate of falls and fall related injury we anticipate preventing functional decline as well as reducing fall-related hospitalisations and the need for escalation in care including a move to a residential aged care facility.

This study will build on extensive pilot work that has identified important risk factors for falls amenable to intervention in older people with CI [1,11-13]. If found to be efficacious in preventing falls, we anticipate the findings will contribute to future guidelines and policies and improve clinical services and the care required for the increasing number of older people with CI across the world.

## Abbreviations

CI: Cognitive impairment; MMSE: Mini-mental state examination; ACE-III: Addenbrookes cognitive examination; FES-I: Falls efficacy scale; IPEQ: The incidental and planned exercise questionnaire; QALYs: Quality adjusted life-years; EQ5D-5 L: EuroQoL 5 dimensions 5-levels; MoCA: The montreal cognitive assessment.

## Competing interests

The authors declare that they have no competing interests.

## Authors’ contributions

All authors contributed to the writing of the grant application for this project which is funded by the Australian National Health and Medical Research Council. All authors contributed to the drafting of the manuscript and approved the final version.

## Pre-publication history

The pre-publication history for this paper can be accessed here:

http://www.biomedcentral.com/1471-2318/14/89/prepub
